# 

**Published:** 1865-10

**Authors:** 


### Books and Pamphlets Received.

LECTURES ON INFLAMMATION: Being the first Course delivered before the College of Physicians of Philadelphia. under the Bequest of Dr. MUTTER. By JOHN H. PACKARD, M. D., Author of "A Manual of Minor Surgery," Translator of Malgaigne's "Traite des Fractures," Secretary of the College of Physicians, etc., etc. Philadelphia: J. B. LIPPINCOTT & Co. 1865.

NATIONAL LYRICS. By JOHN G. WHITTIER. With Illustrations.

SONGS FOR ALL SEASONS, By ALFRED TENNYSON. With Illustrations.

LYRICS OF LIFE. By ROBERT BROWNING. With Illustrations. Boston: TICKNOR & FIELDS. 1865.

